# Multi-sample deformability cytometry of cancer cells

**DOI:** 10.1063/1.5020992

**Published:** 2018-06-21

**Authors:** Shamim M. Ahmmed, Swastika S. Bithi, Adity A. Pore, Noshin Mubtasim, Caroline Schuster, Lauren S. Gollahon, Siva A. Vanapalli

**Affiliations:** 1 Department of Chemical Engineering, Texas Tech University, Lubbock, Texas 79409, USA; 2 Department of Biological Sciences, Texas Tech University, Lubbock, Texas 79409, USA

## Abstract

There is growing recognition that cell deformability can play an important role in cancer metastasis and diagnostics. Advancement of methods to characterize cell deformability in a high throughput manner and the capacity to process numerous samples can impact cancer-related applications ranging from analysis of patient samples to discovery of anti-cancer compounds to screening of oncogenes. In this study, we report a microfluidic technique called multi-sample deformability cytometry (MS-DC) that allows simultaneous measurement of flow-induced deformation of cells in multiple samples at single-cell resolution using a combination of on-chip reservoirs, distributed pressure control, and data analysis system. Cells are introduced at rates of O(100) cells per second with a data processing speed of 10 min per sample. To validate MS-DC, we tested more than 50 cell-samples that include cancer cell lines with different metastatic potential and cells treated with several cytoskeletal-intervention drugs. Results from MS-DC show that (i) the cell deformability correlates with metastatic potential for both breast and prostate cancer cells but not with their molecular histotype, (ii) the strongly metastatic breast cancer cells have higher deformability than the weakly metastatic ones; however, the strongly metastatic prostate cancer cells have lower deformability than the weakly metastatic counterparts, and (iii) drug-induced disruption of the actin network, microtubule network, and actomyosin contractility increased cancer cell deformability, but stabilization of the cytoskeletal proteins does not alter deformability significantly. Our study demonstrates the capacity of MS-DC to mechanically phenotype tumor cells simultaneously in many samples for cancer research.

## I. INTRODUCTION

There is a growing interest in measuring and studying cell deformability, i.e., a cell's ability to deform or change shape under load.^6,7^ Particularly in the context of cancer, investigations of cell deformability are gaining prominence since mechanical and biochemical cues can alter cancer cell deformability, which may in turn influence malignant transformation and tumor growth.^8,9^ From a cancer diagnostics perspective as well, efforts are growing to develop cell deformability as a label-free marker to detect cancer cells in patient samples.^10–12^ Deformability of cancer cells can also be potentially used as a functional readout during compound screening to identify cancer drug candidates.^13,14^

To characterize cancer cell mechanical properties including deformability, numerous techniques have been developed. Popular among these methods are micropipette aspiration,^15,16^ atomic force microscopy,^12,17^ magnetic bead rheology,^18,19^ and optical stretching.^20,21^ These techniques provide reliable mechanical measurements of cells but suffer from low throughput, typically <O(1) cell/s, which is not sufficient to perform large-scale phenotyping of cancer cells and capture the underlying heterogeneity and subpopulations. To address the need for large-scale phenotyping at single cell resolution, researchers have introduced several techniques^1,2,4,22–25^ that exploit the power of microfluidics, advanced image analysis, and electrical readouts.

Recently, image-based large-scale microfluidic single cell mechanical property measurement techniques have emerged that can achieve throughputs of ∼10–1000 cells/s (Refs. 1, 2, 4, and 22) that are broadly referred to here as deformability cytometry (DC)—see Table I. In general, two approaches to deformability cytometry have been developed based on the way deformation is induced on cells. In one class of approaches, cells are driven through the constricted channels of hydraulic diameter smaller than the cell diameter. These constriction-based DC devices either have a single constriction^5,26^ or multiple constrictions interconnected in a parallel network^27,28^ to analyze many cells and achieve higher throughput. In this case, metrics such as cell entry and passage time are measured from images to infer about the deformability of cells.^5,23,26,28,29^ Recently, Lange *et al.*^2^ and Nyberg *et al.*^4^ extracted cell elasticity and fluidity from the measured entry times using a power-law rheology model of cell mechanics. In these studies, cells were deformed at a throughput of ∼O(10) cells/s.

**TABLE I.** Summary of studies that reported image-based large-scale phenotyping of cells.

Study	Geometry	Shear/strain rate (s^−1^)	Cell lines	Throughput (cells s^−1^)
Gossett *et al.*^1^	Cross-slot	∼10^5^	hESC, mESC, HeLa, MCF7, 3T3, PBMCs and Granulocytes	O(1000)
Lange *et al.*^2,3^	Microconstrictions	∼588^a^	MDA-MB231, K562, HEK293T	O(10)
Nyberg *et al.*^4,5^	Microconstrictions	∼170^a^	HL60, MCF7, MDA-MB231	O(10)
Otto *et al.*^22^	Linear channel	∼5 × 10^3^–1.5 × 10^4a^	HL60, whole blood	O(100)
This work	Array of linear channels	∼1.5 × 10^4^–3.5 × 10^4^	MCF10A, HMS50, MCF7, MDA-MB231, MDA-MB468, HCC1419, LNCaP, CL2, PC3	O(100)/sample

^a^ Calculated values.

A significant drawback of constriction-based DC devices is the frictional interaction between the cell and channel walls which can also contribute to the measured cell entry times, in addition to cell deformability.^27^ These wall interactions can also lead to clogging of the device. Especially with an interconnected parallel network of constrictions, clogging in one channel changes the flow rate in the remaining channels affecting the measured readouts. As a result, the constrictions need to be designed optimally and the channel walls need to be treated with anti-fouling agents.

To circumvent the issue of direct interaction of cells with channel walls and clogging, another class of DC techniques have been developed in which either extensional or shear forces are used to deform cells in microfluidic devices. Gossett *et al.*^1^ used a cross-slot geometry coupled with inertial focusing to deform the cells by elongational forces generated at the stagnation point. This technique of hydrodynamic stretching induces significant cell deformations at a throughput of ∼O(1000) cells/s, but requires very high camera frame rates ∼100 000–500 000 per second to capture the images.^30^ As an alternative to the cross-slot geometry, Otto *et al.*,^22^ deformed cells in a linear channel using shear and pressure forces, and implemented real-time image analysis to characterize cell deformability at a throughput of O(100) cells/s.

Existing DC techniques (Table I) have achieved higher throughput by analyzing many cells in a single experiment, however, not much work has been done to configure these approaches to measure the deformability of multiple cell-samples simultaneously. The need to process many samples arises in several cancer-related applications including analysis of cytopathology fluids from patients,^10,11^ screening for anti-cancer compounds,^31,32^ and identifying genes that regulate different steps in the metastatic cascade (e.g., epithelial-mesenchymal transition^33,34^) For example, in cancer drug discovery, a single dose-response curve requires analysis of about 5 samples spanning at least 4 orders of magnitude in dosage. Expanding this to even 10–100 compounds in a pilot screen requires analysis of more than 100 samples, highlighting the need for multi-sample deformability cytometry (MS-DC) techniques.

Advancement of methods that are not only high throughput, but also process numerous samples will bring cell mechanics into the realm of clinical cancer research. Literature reveals two reports of multi-sample evaluation of cell mechanical properties. Qi *et al.*^35^ employed microfiltration using a custom-made well-plate where cells are forced through membrane filters in each well. In contrast, Cribb *et al.*^36^ applied passive microbead rheology to cells cultured in multiwell plates and performed automated analysis to quantify cellular mechanical properties. In both cases, the multiwell format allowed analysis of multiple samples simultaneously.

Examining the available DC techniques for multi-sample deformability analysis, the approach of Otto *et al.*^22^ seems favorable due to the simplicity of the channel geometry. In this work, we build on their approach but use an array of linear microchannels combined with a microfluidic manifold to perform multi-sample deformability cytometry (MS-DC). We use distributed pressure control to simultaneously deform cancer cells in ten independent channels enabling us to run ten samples per experiment, at a throughput of 100 cells/s in each sample. An automated image analysis routine was developed to quantify the deformation index (DI) for every cell in each of the ten channels.

Previously, Otto *et al.*^22^ measured the deformability of HL-60 cells which is a blood-cancer cell line and it is not clear whether tissue cancer cells from solid tumors can be deformed due to flow in linear channels. In this study, we test a panel of cell lines derived from solid tumors, which are usually stiffer than HL-60. The library consists of six breast cancer cells and three prostate cancer cells having different molecular histotype, cellular origin and morphology, and metastatic potential. We also measured the deformability of cancer cells treated with six cytoskeletal intervening drugs. Broadly, our results validate MS-DC as a high throughput platform for mechanical phenotyping of individual cancer cells in multiple cell-samples.

## II. RESULTS

### A. MS-DC device design, set up and manifold characterization

**FIG. 1.**
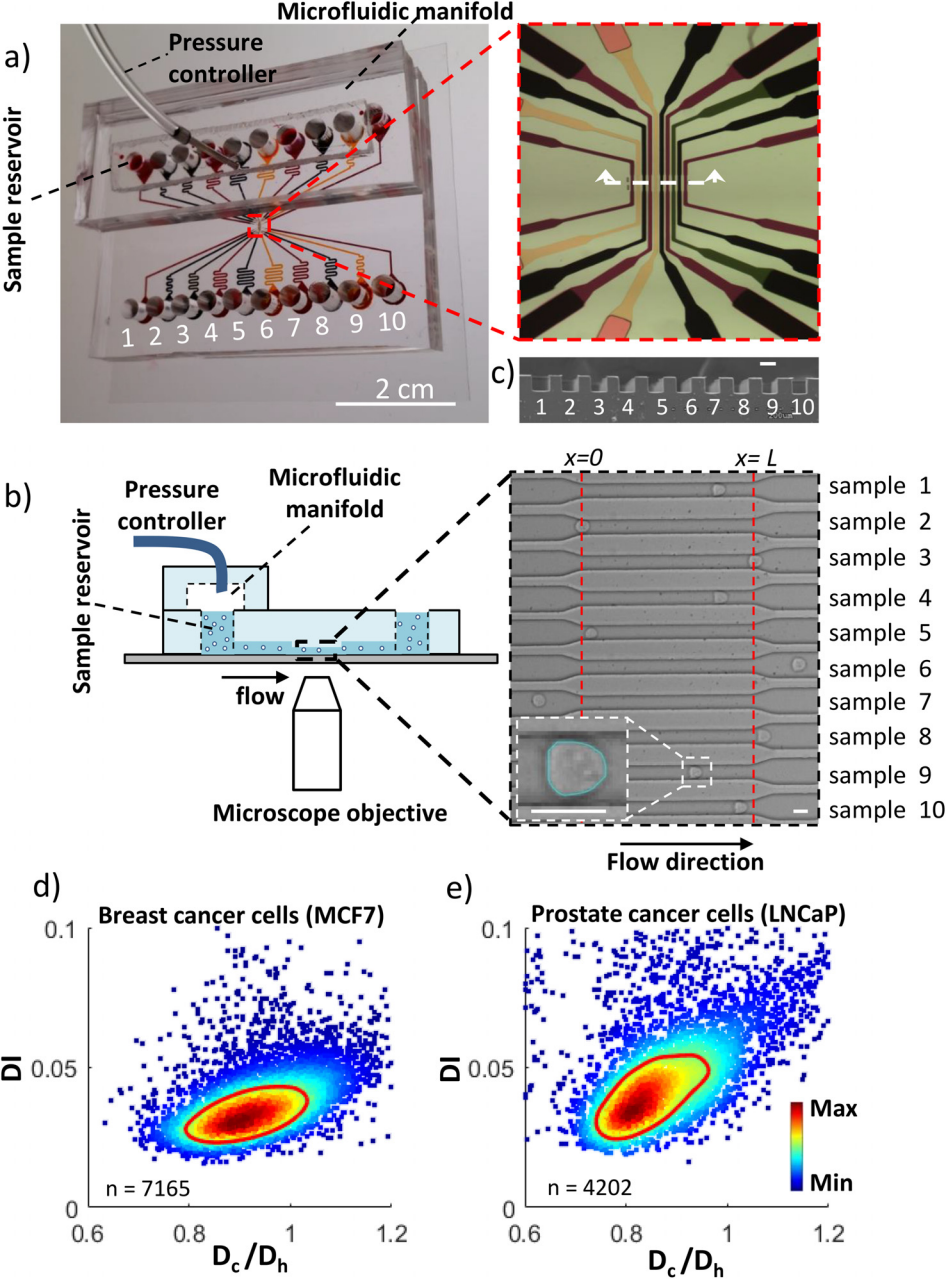
Microfluidic setup for multi-sample deformability cytometry. (a) Image of the microfluidic device showing the microfluidic channel (dyed with different colors), sample reservoirs, outlet from the pressure controller, and microfluidic manifold. The inset shows the zoomed in view of ten independent microchannels. (b) Schematic of the experimental setup. The inset is the bright field image with cells flowing through the channels. The dashed lines represent the length *L* of the test section of the channel. Detected boundary of a deformed cell inside the microchannel is also shown in the bottom left corner of the inset. (c) SEM image of the cross-section of the test channels showing the nearly square cross-section. The scale bar is 18 *μ*m. Scatter density plot of the deformation index (DI) as a function of cell diameter normalized by the hydraulic diameter of the channel of (d) breast cancer cells MCF7 (n = 7164) and (e) prostate cancer cells LNCaP (n = 4202) analyzed from a 5.86 s-long recorded video for each of the cell systems using a driving pressure of 15 kPa. The color indicates the linear density scale and the red line is the contour of the 50% maximal event density.

The MS-DC device design consists of ten independent microchannels [Fig. 1(a)], made using PDMS-based soft lithography techniques.^37^ A PDMS manifold [Figs. 1(a) and 1(b)] chamber was fabricated to distribute the pressure across each on-chip reservoir. The manifold chamber was molded from a 3D-printed master containing a filament structure of height 3 mm, width 7 mm and length 44 mm. The test section of each channel has a width W = 18 ± 0.3 μm, height H = 19.8 ± 0.4 μm and length L = 230 μm, respectively. This almost square cross-section of the test channels is also evident from the scanning electron micrographs shown in Fig. 1(c). To accommodate ten channels with reservoirs in a linear fashion we had to make the channels long. From inlet reservoir to outlet sample collector, the total length of each channel is 43 mm, which offers very high hydrodynamic resistance to the flow. To reduce the resistance due to the long channel, a section of the entry and exit (20 mm each side) were made with a larger cross-section (W = 200 μm & H = 100 μm) than the test section using two step lithography [inset of Fig. 1(a)]. The test section occupied ≈31% of the total channel resistance.

The experimental setup for MS-DC consists of the microfluidic device, a constant pressure source and an inverted microscope connected with a high-speed camera [Fig. 1(b)]. Each of the microchannel accommodates a unique cell-sample. The deformed cells [inset of Fig. 1(b)] were analyzed using custom-written image analysis routines. Figs. 1(d) and 1(e) show the typical scatter density plot of deformation index (DI) versus the ratio of cell diameter to channel hydraulic diameter (D_c_/D_h_), for breast and prostate cancer cells respectively.

**FIG. 2.**
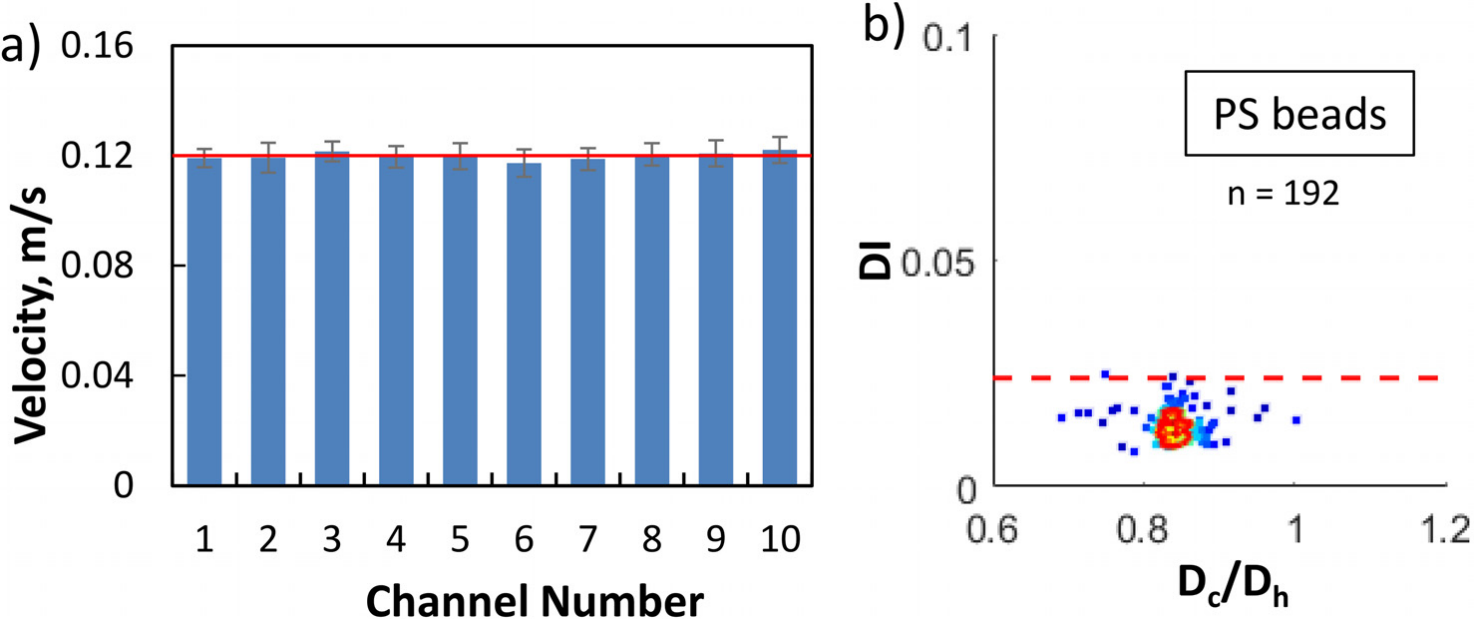
Characterization of the microfluidic manifold and deformability measurements. (a) Mean velocity of monodisperse rigid polystyrene (PS) beads of a mean diameter of 15.13 *μ*m in each of the ten channels for an applied driving pressure of 5 kPa in the microfluidic manifold. The mean velocity was calculated from 20 to 200 particles. The horizontal line is the average of the velocity of PS beads in all the channels and the error bar indicates one standard deviation. (b) Scattered density plot of the deformation index (DI) of PS beads as a function of bead's diameter normalized by the hydraulic diameter of the channel. The red line is the 50% event density contour. The maximum measured DI of PS beads is 0.024. Although most of the beads have lower DI than the maximum value, we take 0.024 as the maximum error in DI measurements in our MS-DC technique.

To asses if the single pressure source and manifold were distributing the same pressure gradient in each of the channels, we measured the velocity of rigid polystyrene (PS) beads (Polyscience Inc., USA) in each of the channel at a driving pressure of 5 kPa. The manufacturer reported bead diameter is D = 15.13 μm ± 6%. As shown in Fig. 2(a), the average velocity of PS beads (calculated from ≥20 beads/channel) varied less than 3% between the channels in the device and therefore the manifold was distributing pressure uniformly across each channel. The slight variations in mean velocity between channels may be due to the variation in bead size as well as the off-centeredness of the particles inside the channel.^38^ We note that since the channels are not interconnected and pressure controller maintains a constant pressure, in case one channel is clogged, the other channels remain unaffected.

### B. Influence of driving pressure on deformation behavior of cells

Our approach of measuring deformability of cancer cells relies on flowing them through the microchannel using a constant pressure drop across the channel. In the microchannel, cells are expected to change their initial spherical shape to bullet-like due to the combination of shear and pressure forces. Since these forces depend on the magnitude of applied pressure drop, we investigated the influence of driving pressure to identify the driving pressures that can induce cell deformation; and how this deformation varies as the cell is moving from the entrance to the exit of the microchannel.

**FIG. 3.**
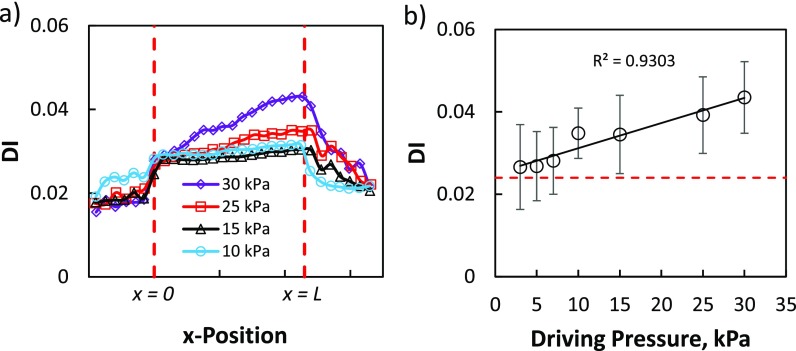
Influence of driving pressure on the deformation behavior of cells in MS-DC. (a) Three point moving average of DI as a function of x-position in the channel for the breast cancer cell line MCF7 at driving pressures 10 kPa (black triangles), 15 kPa (blue circles), 25 kPa (red squares), and 30 kPa (purple diamonds). Each data point on the curves is the mean DI of ≥52 cells taken from all of the ten channels with cell size 0.85 < D_c_/D_h_ < 0.95. The vertical dashed line represents the length *L* of the test section of the channel. (b) DI as a function of driving pressure for the breast cancer cell line MCF7. Each point represents the measured mean DI of the cells (≥52) with the same bin size 0.85 < D_c_/D_h_ < 0.95 and the connected line is the linear fit of the data with R^2^ = 0.9303. The horizontal dashed lines show the maximum error limit in DI measurements.

Figure 3 shows the deformation behavior of breast cancer cells (MCF-7) at driving pressures of 3, 5, 7, 10, 15, 25, and 30 kPa. We observe that before entering the channel, cells had a mean DI less than our maximum error limit of 0.024 for all the driving pressures indicating that they are nearly spherical [Fig. 3(a)]. As the cells pass through the entrance of the channel, for all the driving pressures, the DI jumps to a value higher than the error to comply with the increased fluid stress due to the reduced channel cross-section. For all driving pressures, DI gradually increases after the initial jump to a maximum before cells exit the channel. After exiting the channel, cells tend to relax as their DI decreases.

We find that for all the driving pressures, cells experienced the maximum deformation before exiting the channel. We use this maximum DI before exiting the channel as the measure of their deformability and plot in Fig. 3(b) by binning the cells having confinement 0.85 < D_c_/D_h_ < 0.95, where D_c_ is the undeformed cell diameter and D_h_ is the hydraulic diameter of the channel. The lower limit of the bin was chosen to be 0.85 because below this confinement we did not observe much deformation and the upper limit of the bin was chosen as 0.95 to avoid possible contact between the cell surface and channel wall. Figure 3(b) shows that deformation of MCF7 cells increases linearly with increasing driving pressure.

The results of Fig. 3 also enable us to select a working driving pressure for cancer-related applications. The selection depends on the trade-off between the amount of cell deformation and the volume of images generated. At higher driving, pressure cells deform more but the volume of images generated due to the higher recording frame rate is also high. In this study, we choose 15 kPa as the working driving pressure since this pressure generates enough deformation as well as requires a relatively lower recording frame rate. Also at 15 kPa driving pressure, the cell reaches a steady shape inside the test section of the channel [Fig. 3(a)].

### C. Label-free mechanical phenotyping of metastatic capacity of cancer cells

**FIG. 4.**
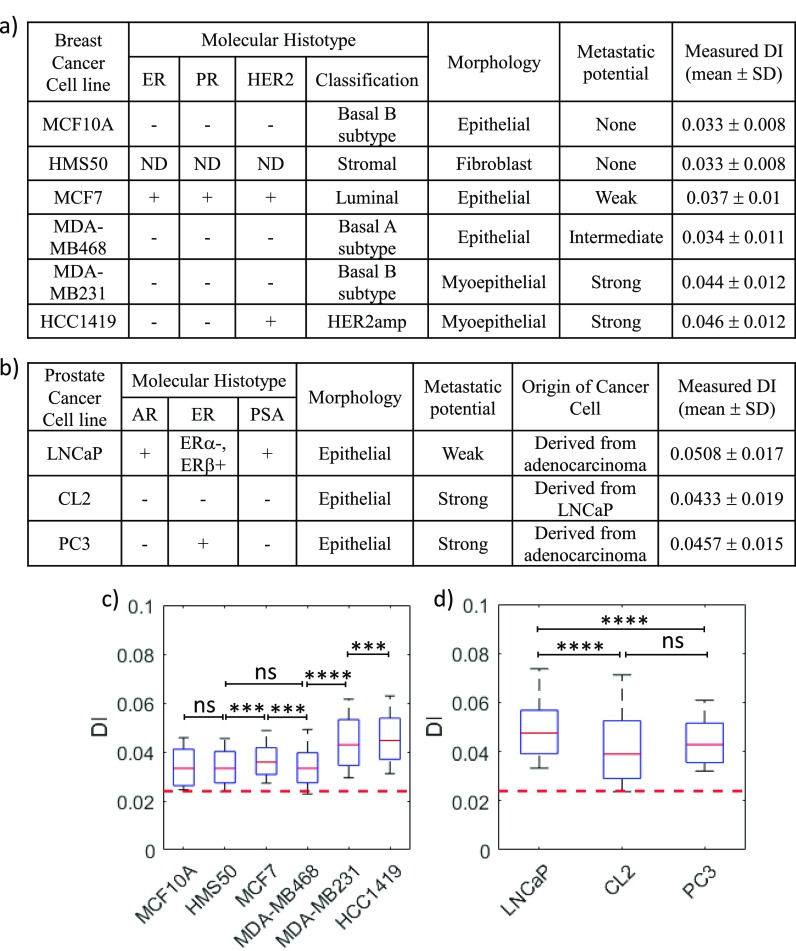
Deformability-based mechanical phenotyping of breast and prostate cancer cells. The metastatic potential, histotype, morphology, origin of the cell lines, and measured DI of six breast cancer cells (a) and three prostate cancer cells (b) are summarized (ER, Estrogen Receptor; PR, Progesterone Receptor; ND, Not determined; AR, Androgen Receptor; and PSA, Prostate Serum Antigen). The boxplots represent the size-gated distribution (0.85 < D_c_/D_h_ < 0.95) of the measured DI of breast cancer cell lines (c) and prostate cancer cell lines (d). The driving pressure is 15 kPa for all the cell lines. The central red line in the box represents the median, the bottom, and the top edges of the box indicate the 25th and 75th percentiles, and the whiskers represent the 10th and 90th percentiles. In the boxplot, n >100 for all the cell lines. Scatter density plots of DI versus cell size are shown in supplementary material Fig. S1 for these cell lines. The statistical significance was determined using the one-tailed Mann-Whitney U test with a significance level of 0.01: ns (p > 0.01); *** (p < 0.0001); and **** (p < 0.00001). The horizontal dashed line in (c) and (d) shows the maximum error limit in DI measurements.

As a first demonstration of the utility of MS-DC, in this section, we present the mechanical phenotyping of six breast cell lines and three prostate cancer cell lines having different metastatic potential, histotype, morphology, and origin [summarized in Figs. 4(a) and 4(b)], using deformability as a label-free marker. This study was conducted by injecting 2–3 different cell lines and their replicates simultaneously in the MS-DC device.

Among the breast cell lines tested, two are non-tumorigenic (MCF10A & HMS50), one is weakly metastatic (MCF7), one has an intermediate metastatic potential (MDA-MB468), and two are strongly metastatic (MDA-MB231 and HCC1419).^39,40^ To compare the deformability of these cancer cell lines, we binned the cells with 0.85 < D_c_/D_h_ < 0.95, and performed the Mann-Whitney U test since the measured deformability has a non-normal (log normal) distribution [Fig. 4(c)]. We find that the strongly metastatic cells (MDA-MB-231 and HCC1419) are much more deformable than both the less-metastatic cells (MCF-7 and MDA-MB468) and normal cells (MCF10A and HMS50). Interestingly, we find that the enhanced deformability of the strongly metastatic cells does not correlate with the histotype, but correlates well with the myoepithelial morphology of these cell types.

In addition to breast cancer cell lines, we tested prostate cancer cell lines: LNCaP, CL2, and PC3 with different metastatic potentials.^41–43^ Here, CL2 is androgen-dependent derivative of androgen-independent LNCaP, hence these two cell lines are isogenic but vary in their metastatic potential.^44^ In contrast to breast cancer cell lines, in the case of prostate cancer cells, we find that the weakly metastatic cells LNCaP are more deformable than the strongly metastatic cells CL2 and PC3. In these cell lines as well, we observed that the deformability does not correlate strongly with the histotype. In sum, using two tissue-specific cancer cell types, we have shown that MS-DC can certainly distinguish highly metastatic cell lines from weakly metastatic and healthy cells, but the correlation between metastatic potential and deformability might be dependent on the cancer type. Thus, the deformability cytometry technique originally developed by Otto *et al.*^22^ can be expanded to cancer cells of solid tumors.

### D. Deformability based drug dose-response analysis

In this section, we present the capability of MS-DC in performing drug dose-response analysis using deformability as the label-free marker. Current drug dose-response analysis approaches rely heavily on viability and other molecular markers. Deformability measures represent whole-cell physiological readouts and can complement molecular marker analysis. Existing deformability methods^1,2,4,22^ do not allow convenient testing of different doses of drugs in the same run. In fact, it is common in cell mechanics literature to pursue investigations with cells that are treated at a single dosage rather than a range of concentrations. Here, we show that due to access to ten independent channels, in the MS-DC approach, drug-treated cells at different doses can be simultaneously deformed in a single run.

**FIG. 5.**
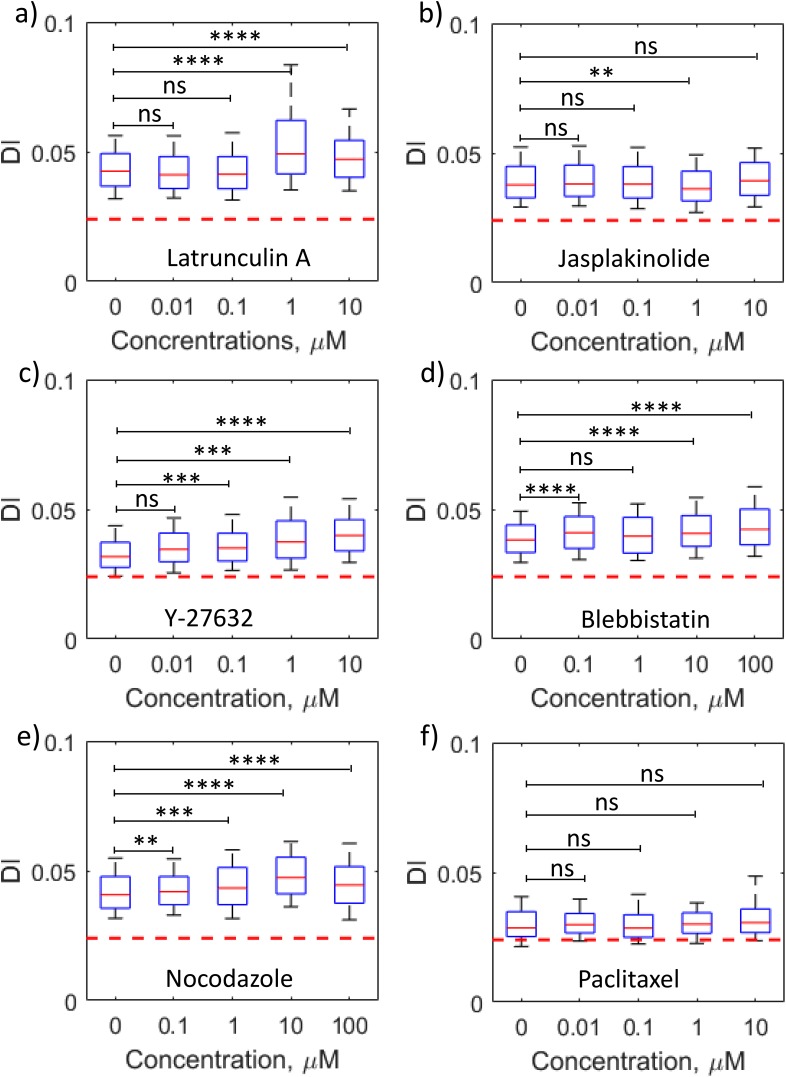
Parallelized drug dose-response analysis of cancer cells using MS-DC. The boxplots represent the size-gated (0.85 < D_c_/D_h_ < 0.95) distribution of measured DI of breast cancer cell line MCF7 treated with actin network intervening drugs (a) Latrunculin A, (b) Jasplakinolide, (c) Y-27632, and (d) Blebbistatin and microtubule intervening drugs (e) Nocodazole and (f) Paclitaxel. The driving pressure is 15 kPa. The central red line in the box represents the median, the bottom, and the top edges of the box indicate the 25th and 75th percentiles, and the whiskers represent the 10th and 90th percentiles. In the boxplot, n ≥ 100 for all the drug concentrations. Representative scatter density plots of DI versus cell size for Latrunculin A are shown in supplementary material Fig. S2. The statistical significance was determined using the one-tailed Mann-Whitney U test with a significance level of 0.01: ns (p > 0.01); ** (p < 0.001); *** (p < 0.0001); and **** (p < 0.00001). The horizontal dashed line in (a)–(f) shows the maximum error limit in DI measurements.

To demonstrate that drug dose-response analysis can be achieved using the ten channel format, duplicates of four different drug concentrations and the control were run simultaneously. A library of six drugs that target the function of cytoskeletal proteins was chosen. Actin-intervention drugs included Latrunculin A, Jasplakinolide, Blebbistatin, and Y-27632. Microtubule-affecting drugs were nocodozole and paclitaxel. The mechanism of action of each of these drugs is listed in Table II. To compare the data for different drug doses for each drug quantitatively, the cells with 0.85 < D_c_/D_h_ < 0.95 were binned and the results are shown in Figs. 5(a)–5(f) (a representative scatter density plot for the *Lat A*-treated MCF7 cell is shown in supplementary material Fig. S2). Overall, it was observed that disruption of the actin network, microtubule network, and actomyosin contractility increased cancer cell deformability, but stabilization of the cytoskeletal proteins did not alter deformability significantly.

**TABLE II.** Summary of deformability response of breast cancer cell MCF7 exposed to actin and microtubule intervening drugs.

Drug	Mechanism of action	Summary of results from MS-DC		
		C_min_ (*μ*M)	Control vs C_min_	C > C_min_
Latrunculin A	F-actin depolymerization	1	DI increases (softening)	Non-monotonic
Jasplakinolide	F-actin polymerization	1	DI decreases (stiffening)	No change
Blebbistatin	Inhibit myosin II activity in actin-myosin network	0.1	DI increases (softening)	Non-monotonic
Y-27632	ROCK inhibitor: inhibit phosphorylation of MLC and Cofilin	0.1	DI increases (softening)	Monotonic increase
Nocodazole	Microtubule depolymerization	0.1	DI increases (softening)	Non-monotonic
Paclitaxel	Microtubule stabilization	…	No change	No change

To quantitate the dose-response behavior, the DI for each drug concentration with control (no drug) was compared and the minimum concentration (C_min_) of the drug was determined as the point at which a change in deformability was observed, i.e., either increase or decrease in DI, as listed in Table II. Blebbistatin, Y-27632, and Nocodazole were observed to be more potent than Latrunculin A since they altered cell deformability at lower dosages, while paclitaxel did not affect cell deformability across the tested range. Thus, MS-DC is capable of identifying drugs and their doses that can alter cancer cell deformability.

To check whether the applied stress in MS-DC had any deleterious effect on the viability of the cells, viability assays were performed. The viability of the to-be-injected cells as well as cells collected from the channel outlets for the drug Latrunculin A was compared [Fig. S3(a)]. The viability was measured for the remaining drugs, after the specified treatment time [Fig. S3(b)]. There was no statistical difference in the viability of injected and collected cells from all five samples [see supplementary material Fig. S3(a)] of Latrunculin A*–*treated MCF7 cells, indicating that the fluid stresses in the channels did not induce any undesirable effect on the cancer cells. For the remaining drugs, the viability was also not affected. Thus, MS-DC is capable of drug dose-response analysis using deformability as a label-free indicator, and it can do so without affecting cell viability.

## III. DISCUSSION

### A. Technical advances of MS-DC compared to other deformability cytometers

There have been two attempts to perform multi-sample characterization of cell mechanics in multiwell plates. In one approach, a microfiltration strategy was used in which cells were forced through membrane filters integrated into a custom multiwell plate.^35^ The fraction of cells passing through the filter in each well are recorded making it a scalable approach with quick population-level readouts. Potential drawbacks of this approach include fouling, lack of readouts on individual cells, and inability to resolve cell-size effects. In another approach, *passive* bead-based microrheology was applied to cells cultured in multiwell plates.^36^ The benefit of this approach is that cancer cells in the adherent state can be interrogated mimicking the tumor microenvironment *in vivo*. The disadvantage of this approach is the lack of single-cell resolution and inadequate control of the attached bead position which makes it difficult to interpret cell rheology from passive bead motion.

Compared to these two prior approaches, this study builds on recent advances in microfluidics-based cell deformability techniques to achieve multi-sample capability. We built our work on the approach of Otto *et al.* since the constriction devices^2,4,28^ are prone to clogging and the extensional deformation of cells requires high camera frame rates^1,45^ which poses an issue for continuous recording and storing images from multiple cell-samples.

In this study, we tested 6 breast cell lines, 3 prostate cell lines, and 6 drugs (×4 concentrations) targeting function. Thus, we have used our multi-sample deformability cytometer to analyze 77 cell samples (including replicates) at rates of 100 cells per second per sample, which could only be possible because of the major improvements we have made. Specifically, (i) Otto *et al.*^22^ use a constant flow rate to deform cells, which is difficult to multiplex, whereas our distributed pressure control allows operation of ten independent channels. (ii) The on-chip reservoirs introduced in our device greatly facilitate multiplexed sample loading, and reduce dead volumes. Sample requirement is at most ∼50 *μ*l, which could be essential for scarcely available rare and patient-derived cells. (iii) Otto *et al.*^22^ report that about 10% of their devices clog, however, MS-DC is inherently fail-safe due to an array of independent channels and our protocol involving two channels per sample. (iv) A data analysis software that tracks cells in each sample and outputs deformed shapes. About 50 000 images were collected from our multi-channel device and processed on a standard desktop computer at a speed of 10 min per sample.

In the future, our MS-DC approach can be reconfigured to deliver new functionalities. For example, rather than using channels of the same width, channels of different width or geometry can be incorporated so that cellular confinement or deformation response is manipulated. In addition, rather than using the same driving pressure, different driving pressures could be integrated into the microfluidic manifold. Such flexibility could be used to obtain multi-dimensional readouts on the same cell sample, which could particularly benefit cancer-related applications given the heterogeneity of tumor cells.

### B. Relationship between cell deformability and metastatic potential

Our study expands the capability of deformability cytometry to simultaneously analyze multiple cell-samples, enabling us to explore the relationship between cell deformability and metastatic potential. With the breast panel, we have chosen cell lines that differ in molecular histotype, morphology, and metastatic capacity. In breast carcinomas, molecular characterization based on ER, PR, and HER2 receptor expression is used to guide decision-making on patient prognosis and treatment.^46^ Our panel of cell lines spanned different molecular sub-types, and we find no strong correlation between cell deformability and molecular sub-type. Fascinatingly, what seems to correlate the best between deformability and metastatic capacity is the cellular origin and morphology of the cells. We find that myoepthelial/basal cells (MDA-MB231 and HCC1419) are more deformable than epithelial-like cells (MCF-7 and MDA-MB468), which is consistent with the basal-like phenotypes having mesenchymal signatures.^47^ Given that breast cancers characterized with HER2 and basal-like phenotypes correlate with poor prognosis and/or resistance to chemotherapy,^48^ suggests that highly deformable breast tumor cells might lead to aggressive cancers. In striking contrast with our study on breast cancer cells where we found that highly deformable cells are more metastatic, in prostate cancer cells, we found the opposite behavior. The reason for this observation is not fully clear although studies have shown that in brain and pancreatic cancer cells,^5,49^ less deformable cells are more metastatic. Thus, the relationship between cancer cell deformability and metastatic capacity needs to be further explored in a broader range of cancers and MS-DC offers a means to pursue such investigations.

## IV. CONCLUSIONS

In this study, we have presented a novel microfluidic technique called multi-sample deformability cytometry (MS-DC) which can measure the deformability of multiple samples simultaneously using a distributed pressure control system and on-chip reservoirs. We have characterized the manifold and standardized the deformability measurements using rigid spherical PS beads. Optimal driving pressure conditions have been identified to adequately deform tissue cancer cells. The MS-DC system was validated by testing breast and prostate cancer cell lines of different metastatic potential and mapping the deformability changes due to different doses of drug treatment. We believe the capacity to process several samples with MS-DC at a throughput of 10 min per sample will be useful in cancer research where there is a need to handle multiple samples for diagnostics, drug discovery, and genetic screens.

## V. METHODS

This study does not require ethics approval.

### A. Cell culture

The breast cancer cell lines HCC1419 (ATCC#CRL-2326); MCF7 (ATCC#HTB-22); MDA-MB231 (ATCC#HTB-26); and MDA-MB468 (ATCC#HTB-132) were obtained from the American Type Cell Collection (ATCC). The non-tumorigenic epithelial cell line MCF10A (ATCC#CRL-10317) was also obtained from the ATCC and was derived from a 36 year-old patient with fibrocystic disease. The normal human mammary stromal (fibroblast) cell line HMS50 was initially developed by Shay *et al.*^50^ and was a kind gift to Dr. Gollahon. The prostate cancer cell lines LNCaP (ATCC#CRL-1740) and PC3 (ATCC#CRL-1435) were obtained from the American Type Cell Collection (ATCC). The prostate cancer cell line CL2 was obtained from Dr. Raul Martinez-Zaguilan.

Breast cancer cell lines MCF7, MDA-MB231, MDA-MB468, and HCC1419 and non-tumorigenic cell line HMS50 were cultured in Dulbecco's modified eagle medium (DMEM) supplemented with 10% fetal bovine serum (FBS, ATCC), 1% Penicillin/Streptomycin (Gibco), and 1% sodium pyruvate (Gibco). Non-tumorigenic breast cell line MCF10A was cultured using DMEM media supplemented with epidermal growth factor (EGF, Peprotch, 1 mg), hydrocortisone (Sigma Aldrich, 1 g bottle), cholera toxin (Sigma Aldrich, 2 mg vials), insulin (Sigma Aldrich, 100 mg vials), and penicillin/streptomycin (Gibco). Prostate cancer cell lines LNCaP and CL2 were cultured using RPMI-1640 media supplemented with 5% FBS, 1% l-glutamine, 1% non-essential amino acid, 1% sodium pyruvate, and 1% penicillin/streptomycin. PC3 cells were also cultured in RPMI-1640 media supplemented with 10% FBS. All the cells were incubated at 37 °C and 5% CO_2_ environment. Confluent cells were harvested for experiment using Trypsin/EDTA (0.25%, Gibco). Trypan blue, at a final concentration of 10% v/v, was added to the suspending phase [phosphate buffered saline (PBS)] to identify dead cells entering to the channel and to have better contrast between cells and the surrounding fluid phase.

In the drug experiments, MCF7 cells were treated with different concentrations of Latrunculin A (Lat A) for 2 h, Jasplakinolide (Jasp) for 1 h, Blebbistatin (Bleb) for 1 h, Y-27632 for 30 min, Nocodazole (Noco) for 1 h, and Paclitaxel (Pac) for 1 hr. All the drugs were dissolved in dimethyl sulfoxide (DMSO) to prepare the stock solution. The required concentrations of the drug were made by diluting the stock solution using PBS. In all cases, the percent of DMSO in the final cell solution was <0.1%, thus avoiding the toxicity from DMSO. Drug solution in PBS was freshly prepared before each experiment and used on the same day. Prepared drug solutions were added to a suspension of 2.25 × 10^5^ cells/ml in cell media in a 12 well plate and incubated at 37 °C and 5% CO_2_ environment. After the specified hours of incubation, cells were trypsinized and cell media with drug was removed as a supernatant by centrifugation. The final cell solution for the experiment was prepared using PBS. A cell concentration of 1.5 × 10^6^ cells/mL was used in all cell experiments. All the experiments were completed within 30 min of cell harvesting.

### B. Microfluidic device preparation

The MS-DC master mold was made using SU-8 photolithography. Polydimethylsiloxane (PDMS) monomer and the curing agent were mixed at a 10:1 ratio, degassed, poured on the master mold to get an approximate 6 mm height, and cured in an oven for at least 2 h at 70 °C. Cured PDMS was cut, peeled off from the mold, and 3 mm diameter inlet and outlet holes were punched using a biopsy puncher (Miltex, Japan). The Inlet holes were used as on-chip sample reservoirs and the outlet holes facilitated the collection of the samples from each channel. Ten holes were punched into the PDMS manifold to introduce samples into the on-chip reservoirs and subsequently all but one were blocked by pins to connect the pressure source.

We clean the PDMS replica, PDMS manifold, and cover glass (No. 2, Fisher Scientific, 45 × 50 mm) using isopropanol for bonding. Bonding is done in two steps. First, we bond the PDMS replica with the cover glass by activating the surfaces with air plasma for 1.5 min (Plasma Cleaner PDC-32G, Harrick Plasma) then we bond the PDMS manifold on top of the inlet reservoir holes using air plasma again. In the second step of bonding, we ensure that the chamber of the manifold is aligned well so that all the on-chip reservoirs are open to the chamber which facilitates the distribution of driving pressure to each reservoir. The entire assembly was baked again at 70 °C for 4 min to have a permanent seal. The sealed device is then filled with PBS to maintain the hydrophilicity of the microchannels.

### C. Experimental protocol

The experimental setup for MS-DC consists of the microfluidic device, a constant pressure source, and an inverted microscope connected with a high-speed camera. Samples were loaded using a 25-gauge hypodermic needle (Excel International disposable) into each of the reservoirs (∼50 *μ*l/reservoir) through the holes punched on the roof of the manifold chamber and then all but one hole on the roof of the manifold were closed using 1 mm diameter pins (Instec, USA). The reservoirs were loaded to 85% of the maximum capacity to avoid the overflow of the cell samples and thus the possibility of any cross-contamination. A constant driving pressure was applied through the open hole across the device using a pressure controller (MFC8-FLEX4C, Fluigent Inc.) and 0.02 in. inner diameter Tygon tubing (Cole Parmer) connected with 20-gauge hollow blunt pin (Instech, USA). A constant driving pressure of 15 kPa was applied for all experiments unless noted otherwise.

High-speed video imaging was conducted to track the cell shape at a single cell level as it passes through the test section of the channel using a combination of an inverted microscope (Nikon eclipse TiU) and a high-speed CMOS camera (Phantom v710 12-bit, Vision Research). The region of interest (ROI) was recorded with a reduced resolution of 464 × 504 pixels at a frame rate of 15 000 fps using 20× magnification. The ROI included the area of test section and five channel-widths before and after the test section. The effective pixel size for this optical setup is 0.97 *μ*m and the depth of focus is ∼6 *μ*m. An exposure time of 1 *μ*s was used to record blur-free motion [inset of Fig. 1(b)] of cells traveling at velocity ∼0.3 m/s. We measured the deformability of MCF7 cells using 20× and 30× magnifications in the same experiment to check whether the higher magnification improves statistical accuracy. The effective pixel sizes for these two magnifications are 0.97 *μ*m and 0.64 *μ*m, respectively. The results (supplementary material, Fig. S4) show that the lower magnification used in this study does not significantly affect the accuracy of the deformability metric.

### D. Image processing

The recorded images were analyzed off-line using a custom-written MATLAB routine capable of automated image processing and data analysis. The images were segmented to have a binary image using different filters to enhance contrast, subtract background, and identify the presence of a cell. After segmentation, each cell was tagged with an identification (ID) number when it appears in the ROI for the first time. Using the MATLAB built-in function “regionprops”, the cell's projected area, centroid location, perimeter, and frame number were recorded against that ID number for all the frames that cell took to pass through the ROI. The undeformed diameter of each cell was calculated from the projected area before it enters into the constricted channel.

To capture the change in cell's shape, the deformation index (DI) was calculated for each cell which was defined as^22^

$$DI=1-\;\frac{2\sqrt{\pi A}}{p}$$

where *A* is the cross-sectional area and *p* is the perimeter. The DI of a perfect sphere is zero. As the cell deviates from the spherical shape, the value of DI increases from zero accordingly. Image frames with multiple cells in the test section of the channel were discounted due to potential hydrodynamic interactions between cells which can interfere with the deformation behavior of cells. To determine the minimum DI that can be reliably measured with our set up, we used rigid spherical polystyrene beads and the resulting DI distribution is shown in Fig. 5(b). We find that the beads show a maximum DI of 0.024 as shown by the horizontal dashed line in Fig. 5(b). Therefore, DI = 0.024 was used as the maximum error in the measurement of DI of cells. In addition, the measured bead diameter was found to be 15.83 *μ*m ± 4%, which is close to the manufacturer reported size distribution of 15.13 *μ*m ± 6%.

## SUPPLEMENTARY MATERIAL

See supplementary material for effects of magnification on the measured deformability, scatter density plots, and viability assays.
